# The complexity of ToxT-dependent transcription in *Vibrio cholerae*

**Published:** 2011-02

**Authors:** Gregor G. Weber, Karl E. Klose, Klose

**Affiliations:** *South Texas Center for Emerging Infectious Diseases & Department of Biology, University of Texas San Antonio, San Antonio TX, USA*

**Keywords:** AraC family, cholera, pathogenicity island, transcription activation, virulence

## Abstract

*Vibrio cholerae* is the causative agent of the disease cholera, characterized by profuse watery diarrhoea. Two of the main virulence factors associated with the disease are cholera toxin (CT) and toxin-coregulated pilus (TCP). Expression of CT and TCP is regulated via a complex cascade of factors that respond to environmental signals, but ultimately ToxT is the direct transcriptional activator of the genes encoding CT and TCP. Recent studies have begun to unveil the mechanisms behind ToxT-dependent transcription. We review current knowledge of transcriptional activation by ToxT and the environmental stimuli that allow ToxT to regulate virulence gene expression, resulting in cholera pathogenesis.

## Introduction

*Vibrio cholerae* is the aquatic bacterium responsible for the disease cholera. In addition to being well-equipped to survive various environmental conditions, *V. cholerae* is also capable of infecting humans, resulting in substantial loss of fluid in the form of diarrhoea and frequently leading to death due to dehydration, if left untreated. The human intestine provides the ideal conditions for the expression of virulence factors that allow the organism to colonize, cause disease, and prepare for re-entry into the environment. The two main virulence factors are cholera toxin (CT) and toxin-coregulated pilus (TCP). CT is an ADP-ribosylating toxin composed of two subunits that causes an increase in cAMP in intestinal cells, leading to diarrhoea due to the osmotic imbalance^1^. The genes encoding CT, *ctxAB*, are encoded on a lysogenic filamentous bacteriophage, CTXΦ^2^ and are under the direct control of the regulatory factor ToxT.

**Fig. 1:**
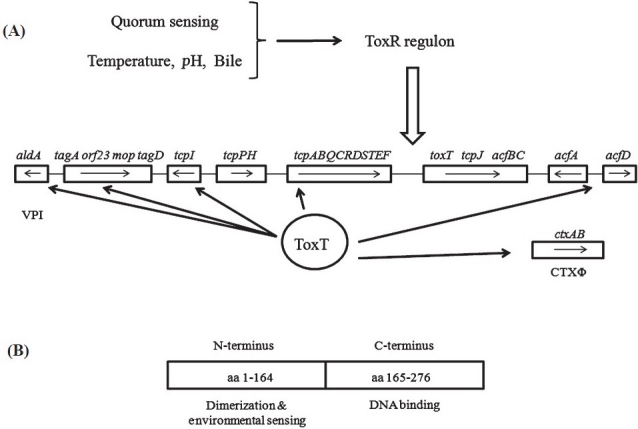
**(A)**. The ToxR regulon. The ToxR regulon is responsible for activating transcription of *toxT*, which facilitates virulence gene expression. Environmental factors such as temperature, bile and *p*H modulate the ToxR regulon. ToxT is the direct transcriptional activator of the *ctxAB* (located in the CTXΦ), *tcp, acfA, acfD, tcpI, aldA* and *tag* genes (all located in the VPI), and it also responds to environmental signals to modulate expression of these genes. **(B)**. ToxT is an AraC-like protein composed of two domains. The N-terminal domain, comprised of amino acids (aa) 1 to 164, is involved in dimerization and environmental sensing, while the C-terminal domain (aa 165 to 276) is responsible for DNA binding.

TCP is a type IV bundle-forming pilus that is critical for intestinal colonization. The *tcp* genes are located on the Vibrio pathogenicity island (VPI), and are under the direct control of ToxT. The *toxT* gene is also encoded within the VPI, as are additional ToxT-dependent factors, such as the accessory colonization factor (ACF) genes (*acfA, acfB, acfC, acfD*, Fig. 1). Disruption of any of these four genes results in a colonization defect within the infant mouse^3^. The function of the various ACF proteins is not well characterized, with the exception of AcfB. AcfB, like another ToxT-dependent VPI protein TcpI, encodes a methyl-accepting chemotaxis protein (MCP)^4^. Deletion of both *acfB* and *tcpI* leads to a decrease in colonization^5^, suggesting that these ToxT-dependent MCPs facilitate chemotaxis to the correct intestinal location for productive colonization. Reviews have been published earlier on the ToxR regulon^6^ discussing as to how environmental signals induce *toxT* transcription. This review will focus on ToxT-dependent transcription, highlighting the complexity of virulence gene expression in *V. cholerae*.

## ToxT domain structure and function

A large amount of research from a number of laboratories has elucidated much of the signaling cascade that results in the expression of CT and TCP. This cascade is frequently referred to as the ToxR regulon. In this regulon, various environmental factors influence a number of regulatory proteins (including ToxR) and this ultimately culminates in the transcription of *toxT*.

ToxT, an AraC-like activator protein, directly activates transcription of the *ctxAB, tcpABQCRDSTEF, tcpI, tagA orf23 mop tagD, aldA, acfD* and *acfA* operons^7^ (Fig. 1A). Because the *toxTtcpJacfBCtagE* operon is located downstream of the tcpA operon, ToxT also autoregulates its own expression as well as that of the other genes in this operon, which are also under the control of the ToxR-TcpP-dependent promoter immediately upstream of the *toxT* gene^8^. Thus, with the exception of the *tcpPH* genes, the entire VPI is under the regulatory control of ToxT, emphasizing the importance of this virulence regulator to *V. cholerae* pathogenesis.

ToxT is composed of two domains: the N-terminus is involved in dimerization and environmental sensing, while the C-terminus contains two helix-turn-helix (HTH) motifs that facilitate binding to the promoter regions of the ctx, tcp, and acf genes^9^–^11^ (Fig. 1B). Comprehensive scanning alanine mutagenesis of ToxT helped to identify specific residues involved in dimerization, DNA binding and transcriptional activation^10^. Residue F151, which lies in a putative alpha helical region of the N-terminus, is critical for dimerization, while residues within HTH1 and HTH2 within the C-terminus failed to activate virulence gene expression highlighting their potential in DNA binding. A computer threading model of the ToxT C-terminus with the AraC family protein MarA predicts that residue T253 in HTH2 makes base-specific contact with the ToxT binding site, which is supported by our unpublished data. Interestingly, various alanine substitution mutants also exhibited differential activation of the *ctx, tcp*, and *acf* genes, indicating that modulation of ToxT may allow for such differential activation *in vivo*. This is supported by previous findings that *tcp* gene transcription precedes *ctx* gene transcription *in vivo*, despite both being activated by ToxT^12^.

## DNA binding by ToxT

**Fig. 2:**
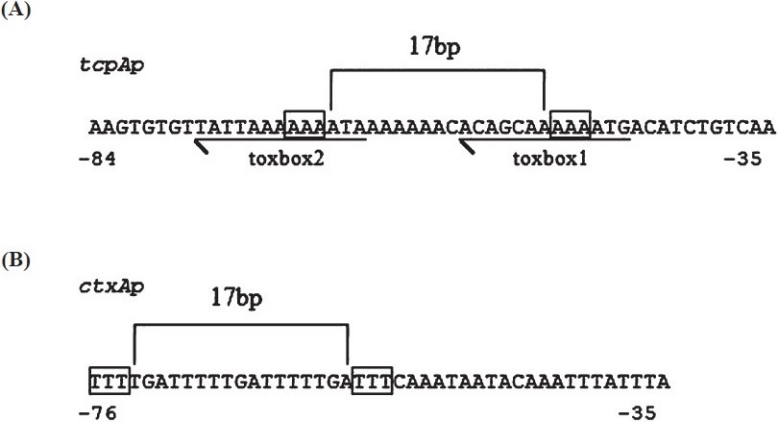
ToxT–activated promoters. A schematic representation of the *tcpA* and *ctxA* promoter regions to which ToxT binds to activate transcription. **(A)**. The *tcpA* promoter contains two “toxboxes” (underlined). The “A/T” triplets within the boxes are critical nucleotides for ToxT-dependent transcription. **(B)**. The ctxA promoter represented in this figure is sufficient for ToxT-dependent transcriptional activation and therefore should contain two “toxboxes”. The boxes outline “A/T” triplets that are spaced identically to the triplets in the *tcpA* promoter, which suggests that ToxT is oriented in the opposite direction with respect to RNA polymerase in *ctxA* promoter as compared to the *tcpA* promoter

Recent work has begun to clarify how ToxT binds to the promoter regions of ToxT-activated genes. ToxT binds to a 13-bp DNA sequence that has been labeled a “toxbox” (yrTTTTwwTwAww)^13^. Two “toxboxes” are positioned as direct repeats in the *tcpA, ctxAB* and *tcpI-2* promoters, and as inverted repeats in the *acfD/acfA, tagA* and *tcpI-1* promoters (two separate binding sites have been identified in the tcpI promoter region). Interestingly, the aldA promoter appears to only contain one toxbox^13^. Mutational analysis of the two toxboxes within the *tcpA* promoter revealed 10 nucleotides within these sequences that are critical for ToxT-dependent transcription, and notably the “T” triplet found in each toxbox was important (Fig. 2A). These triplets are spaced 17 bp apart, which is similar to the spacing between triplets found in the *ctx* (Fig. 2B) promoter that lie within the region bound by ToxT, suggesting that specific protein-DNA contacts may be made in these triplets. The *ctxA* promoter region from -76 to +1 is sufficient for ToxT-dependent transcription. Although similar analysis of the specific basepairs required for ToxT-dependent transcription has not been performed at the *ctx* promoter, we have noted the similarity in “T” triplet spacing (Fig. 2), which would require ToxT to be bound in the opposite orientation with respect to RNA polymerase (due to the opposite orientation of toxboxes) in comparison with the *tcpA* promoter.

Separating the toxboxes in the *tcpA* promoter by 5bp or 10bp disrupted ToxT-dependent transcription, despite evidence that ToxT could still protect these sites in footprinting assays performed *in vitro*^13^. Likewise, insertions between the toxboxes of the *acfA/acfD*, as well as the *tagA* promoters disrupted transcriptional activation of these genes^14^. These data indicate that interaction between the ToxT proteins bound at each site is critical for transcriptional activation. Our own data have supported the idea that ToxT requires dimerization in order to bind to the *tcpA, ctxA*, and *acfA* promoter sites^10^,^11^, which is consistent with the findings of other laboratories^9^. We suggest that the apparent binding of ToxT to the *tcpA* toxboxes separated by 5 and 10 bp is due to the high concentration of ToxT in *in vitro* footprinting reactions, and that *in vivo* ToxT must be dimerized to bind to these sites. However, other sites that ToxT binds may be bound by its monomeric forms.

The spacing between the ToxT binding sites and the -35 region bound by RNA polymerase (RNAP) is also critical for transcriptional activation^14^. The *tcpA, acfA, acfD*, *tagA* and *aldA* promoters were only able to tolerate slight changes in spacing (± 1 to 2 bp) between the proximal toxbox and the -35 region of RNAP. Since the “toxboxes” are all located upstream of the -35 promoter region, this places all ToxT-dependent promoters under the class I promoter category of AraC family activated promoters. This suggests that ToxT directly interacts with the α subunit C-terminal domain (α-CTD) of RNAP to activate transcription, which was supported by transcription experiments performed with ToxT and a α-CTD deletion mutant of RNAP^15^.

## Factors that affect ToxT-dependent transcription

Nucleoid-associated proteins (H-NS) bind to DNA and have the capability of altering its structure, which in turn can affect transcription. In *V. cholerae*, a deletion of *hns (vicH*) resulted in high level expression of the *tcpA* and *ctx* genes, even under non-inducing conditions^16^. High levels of ctx and *tcpA* expression were also observed in an *hns* mutant lacking ToxT. These data indicate that H-NS binds these promoters to repress virulence gene expression in the absence of ToxT. Under inducing conditions, H-NS repression at the *ctx* and *tcpA* promoters can be overcome by ToxT displacement of H-NS^16^.

Integration host factor (IHF) has been shown to positively regulate expression of *tcpA* and *ctx*^17^. IHF is a heterodimeric protein encoded by *ihfA* and *ihfB*, which causes sharp bends upon binding to DNA. The deletion of these two genes resulted in approximately five-fold reduction in *ctx* and *tcpA* expression. It has also been demonstrated that IHF binds at the -162 region of the *tcp* Apromoter and bends the DNA. This region also overlaps with the binding region of H-NS, allowing IHF to displace H-NS, which in turn permits virulence gene expression. However, IHF has not been shown to bind the ctx promoter. Presumably, positive ToxT-dependent regulation at the *tcp*A promoter leads to increased levels of ToxT, which in turn enhances ctx expression^17^.

cAMP-CRP similar to H-NS, also negatively regulates the expression of CT and TCP under certain environmental conditions. However, these are due to effects on the ToxR regulon leading to ToxT expression, rather than effects on ToxT-dependent transcription^18^,^19^. Cyclic diguanylate (c-diGMP) levels influence transcription of ctxA, but surprisingly do not seem to affect *tcpA*A expression. Because low levels of c-diGMP favour ToxT transcription as well as ctx transcription, it is not clear whether c-diGMP specifically affects ToxT-dependent transcription at the ctx but not *tcpA*A promoter or whether this phenomenon only involves ToxT expression but not ToxT activity^20^.

## Environmental factors that directly affect ToxT

Initial studies showed that environmental signals are able to modulate ToxT activity. Both variations in temperature and/or the presence of bile could reduce or eliminate ToxT-dependent transcription of *ctxA, tcpA*A and several other genes^21^,^22^. Further studies on the effects of bile on *V. cholerae* virulence factor transcription revealed that unsaturated fatty acids (arachidonic, linoleic, and oleic acids) within bile repressed *ctxAB* and *tcpA*A expression^23^, suggesting that these components may be directly interacting with ToxT to repress its activity. The presence of high levels of bile within the intestinal lumen may prevent premature ToxT activity prior to the organisms’ arrival within intestinal crypts. Anaerobiosis was also found to specifically inhibit *ctxA* transcription, but this effect is likely to be mediated by the effect on H-NS binding, rather than the effect on ToxT activity^24^.

A synthetic small molecule, virstatin, was identified for its ability to inhibit ToxT-dependent transcription^25^. Virstatin inhibits the dimerization of the N-terminus of ToxT, and thus prevents ToxT-dependent transcription activation of the ctxAB, *tcpA, acfD* and *tagA* promoters^9^,^26^. It is tempting to speculate that virstatin is a synthetic mimic of the effect of bile components on ToxT.

Bicarbonate has the opposite effect of bile/virstatin on ToxT activity. Low levels of bicarbonate result in low ToxT dependent transcription of *tcpA* and *ctxA*, whereas elevated levels of bicarbonate lead to maximal transcription of *ctx* and *tcp*. It is suggested that bicarbonate is found at high concentrations in the intestinal epithelium and, thus, may act as a relevant *in vivo* signal modulating the activity of ToxT^27^. From these collective data, it is evident that ToxT activity is modulated by various environmental signals, and that the protein receives signals either indirectly or directly through binding of effector molecules.

## ToxT as a repressor of gene expression

The type IV mannose-sensitive haemagglutinin (MSHA) contributes to *V. cholerae* biofilm formation and persistence in the aquatic environment, but it is repressed during *in vivo* growth by ToxT. Repression of MSHA allows *V. cholerae* to evade binding of sIgA and establish residence within the host^28^. ToxT inhibits *mshA* transcription by binding to three binding sites within the *msh* operon^29^. Interestingly, ToxT dimerization is not required for repression of *mshA* transcription; ToxT either containing a dimerization point mutation, or lacking the entire N-terminus was able to repress *msh* transcription^30^. As mentioned earlier, dimerization mutants fail to activate the ctx and *tcp* genes, suggesting that ToxT binding sites can be differentiated between those that require dimerization for binding (ToxT-activated promoters) and those that can bind monomeric ToxT (ToxT-repressed promoters). One notable exception appears to be the ToxT-activated aldA promoter, which appears to only bind a monomer of ToxT^13^,^14^,^31^.

## Conclusion

ToxT-dependent transcription is a critical component of *V. cholerae* pathogenesis. ToxT is able to differentially regulate virulence gene expression by directly binding to degenerate promoter DNA sequences. Available data imply that ToxT needs to dimerize in order to activate genes (*ctxA, tcpA, acfA, acfD, tcpI*), but it can bind as a monomer to repress (*msh*) and, possibly, even activate (*aldA*) other genes. ToxT binds to “toxboxes” that is oriented differently at the various promoters. This suggests a model whereby ToxT shows flexibility in its DNA binding capabilities and interactions with RNAP. ToxT activity is modulated by environmental signals resulting in optimal temporal and spatial virulence factor expression within the host. Further research is required to fully characterize the complexity of ToxT-dependent virulence gene expression in *V. cholerae.*
